# Understanding negative feedback from South Asian patients: an experimental vignette study

**DOI:** 10.1136/bmjopen-2016-011256

**Published:** 2016-09-08

**Authors:** Jenni Burt, Gary Abel, Natasha Elmore, Cathy Lloyd, John Benson, Lara Sarson, Anna Carluccio, John Campbell, Marc N Elliott, Martin Roland

**Affiliations:** 1 Cambridge Centre for Health Services Research, Institute of Public Health, Forvie Site, University of Cambridge School of Clinical Medicine, Cambridge, UK; 2 University of Exeter Medical School, Exeter, UK; 3 Faculty of Health and Social Care, The Open University, Milton Keynes, UK; 4 Primary Care Unit, Institute of Public Health, Forvie Site, University of Cambridge School of Clinical Medicine, Cambridge, UK; 5 Ipsos MORI, London, UK; 6 University of Exeter Medical School, Exeter, UK; 7 RAND Corporation, Santa Monica, California, USA

**Keywords:** communication, healthcare disparities, minority groups, physician-patient relations, PRIMARY CARE

## Abstract

**Objectives:**

In many countries, minority ethnic groups report poorer care in patient surveys. This could be because they get worse care or because they respond differently to such surveys. We conducted an experiment to determine whether South Asian people in England rate simulated GP consultations the same or differently from White British people. If these groups rate consultations similarly when viewing identical simulated consultations, it would be more likely that the lower scores reported by minority ethnic groups in real surveys reflect real differences in quality of care.

**Design:**

Experimental vignette study. Trained fieldworkers completed computer-assisted personal interviews during which participants rated 3 video recordings of simulated GP–patient consultations, using 5 communication items from the English GP Patient Survey. Consultations were shown in a random order, selected from a pool of 16.

**Setting:**

Geographically confined areas of ∼130 households (output areas) in England, selected using proportional systematic sampling.

**Participants:**

564 White British and 564 Pakistani adults recruited using an in-home face-to-face approach.

**Main outcome measure:**

Mean differences in communication score (on a scale of 0–100) between White British and Pakistani participants, estimated from linear regression.

**Results:**

Pakistani participants, on average, scored consultations 9.8 points higher than White British participants (95% CI 8.0 to 11.7, p<0.001) when viewing the same consultations. When adjusted for age, gender, deprivation, self-rated health and video, the difference increased to 11.0 points (95% CI 8.5 to 13.6, p<0.001). The largest differences were seen when participants were older (>55) and where communication was scripted to be poor.

**Conclusions:**

Substantial differences in ratings were found between groups, with Pakistani respondents giving higher scores than White British respondents to videos showing the same care. Our findings suggest that the lower scores reported by Pakistani patients in national surveys represent genuinely worse experiences of communication compared to the White British majority.

**Strengths and limitations of this study**

- This is the first study to exclusively use a video vignette approach to assess the extent to which ethnic differences in reported patient experience of primary care reflect real differences rather than differences in expectations, perceptions or in the use of scales.
- Our experimental design enables us to control the content of the consultations being rated by respondents in order to explore how differences in reporting may or may not explain the disparities in minority ethnic experience in real-life surveys.
- While our in-home face-to-face recruitment approach ensured access to a wide range of respondents, respondents who agreed to participate in this study may differ in a number of unidentified ways from the population as a whole.
- Our study involved face-to-face interviews in which consultations were viewed and rated: this differs from the postal mode of the national GP Patient Survey, and in completing questionnaire items via an interviewer rather than independently, social desirability bias may be an issue.
- To enable the same vignettes to be viewed by all participants, the study was conducted in English, limiting our ability to understand evaluations by those with low English language proficiency.

## Introduction

Communication between doctors and patients is a core component of patient experience.^1^ Patients' evaluations of doctors' interpersonal skills are widely used in assessments of the quality of care, with an increasing focus on the public reporting of patient feedback.^2^ In the USA and the UK, certain minority ethnic groups report lower patient experience scores compared to the majority population.^3–8^ For example, analysis of the English General Practice Patient Survey found that South Asian groups report particularly low scores compared to the White British majority, with Bangladeshi and Pakistani groups providing the lowest scores.^9^ Around half of the difference in these scores is explained by the concentration of South Asian patients in low-scoring primary care practices.^7^ The remaining difference currently remains unexplained.

Several potential explanations have, however, been proposed for the lower ratings given by South Asian respondents. Broadly, these relate to whether South Asian patients receive lower quality care, or whether they receive similar care, but rate this more negatively.^8^ For example, differences in the use of questionnaire response scales^10^ may lead to South Asian groups being less likely to endorse the most positive options when asked to evaluate a doctor's communication skills. Alternatively, there may be systematic variations in evaluations of consultations because South Asian respondents vary in their expectations of, or preferences for, care.

Understanding why minority ethnic groups often give poorer evaluations of care is critical to forming an effective response in policy and practice. One approach is to use item response theory to explore whether items receive systematically different responses by the ethnic group. Recent analysis of the GP Patient Survey suggests that this is not the case for differences observed between South Asian and White British groups.^11^ However, a more robust approach to determining whether differences in evaluations of care reflect real differences is to ask respondents to rate standardised clinical scenarios (‘vignettes’).^12^ A US study using primarily written vignettes in an online survey concluded that score variations observed on national surveys among African-American, Latino and white respondents were likely to reflect true differences in real-life experiences, at least for items using an ‘Always-to-Never’ Response Scale.^10^ ^13^

This study aimed to examine whether people from a Pakistani background rate the communication within simulated GP consultations differently than White British people do. If these groups rate simulated consultations similarly when viewing identical video vignettes, it is more likely that the lower scores observed among South Asian people in national patient experience surveys reflect real differences in quality of communication within consultations.

## Methods

We undertook an experimental vignette study in which videos of simulated GP–patient consultations were shown to two groups of people, who were asked to rate the quality of the communication within each consultation. The primary outcome of interest was communication score (on a scale of 0–100).

### Simulated consultations

**Figure 1:**
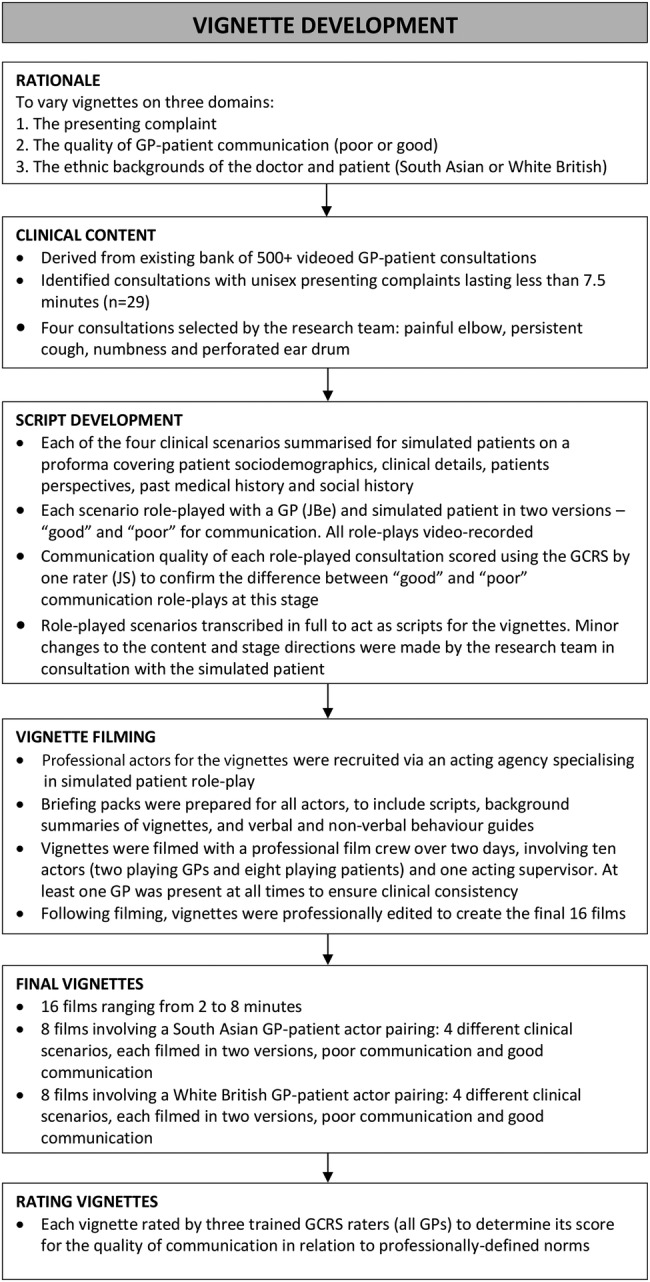
The vignette development process.

To ensure generalisability and to avoid the chance inclusion of a characteristic or event which, unknown to us, might systematically be rated differently by our two groups of participants, we produced a series of 16 vignettes for this study. We sought to manipulate the vignettes on three key domains: (1) the presenting symptom; (2) the quality of the communication within the consultation (poor or good) and (3) the ethnic backgrounds of the doctor and patient (South Asian or White British). Following published recommendations for the production of vignettes, we based ours on real-life GP–patient consultations recorded as part of another study.^14^ We undertook an extensive process of script development, roleplaying and rating prior to filming the vignettes with professional actors (figure 1).

Vignettes covered four different clinical scenarios: persistent cough, perforated ear drum, painful elbow and generalised numbness. We devised two different scripts for each scenario: one designed to illustrate poor communication by the doctor, and one designed to illustrate good communication. Standards of communication were formulated according to the Global Consultation Rating Scale (GCRS), an observer-rated measure of communication competence derived from the widely used Calgary-Cambridge guide to the medical interview.^15–17^ The GCRS covers 12 domains including ‘initiating the session’, ‘gathering information’, ‘building the relationship’ and ‘achieving a shared understanding’. The ‘poor’ and ‘good’ versions of the four clinical scenarios were then used to film two sets of vignettes, first with White British actors playing the GP and patient, and then with South Asian actors playing the GP and patient. The GP role was acted throughout by either one White British or one South Asian actor; eight different actors (four White British and four South Asian) role-played patients, each participating in one clinical scenario. The restriction of vignettes to the same-ethnicity pairings, rather than including mixed pairings, is a function of wishing to introduce some variation to ensure generalisability while keeping the number of vignettes to a manageable number. The final 16 videos were each scored by 3 clinical raters using the GCRS to assess communication quality in relation to professionally defined norms.^15^ The ‘poor’ communication vignettes had mean GCRS scores between 0.6 and 2.4 (out of 10) while the ‘good’ communication vignettes mean scores were between 5.1 and 8.4.

### Data collection

We worked with a UK market research company, Ipsos MORI, to collect the data. We aimed to recruit 1120 adult respondents who self-identified as either Pakistani or White British, across a broad age range. Each respondent was asked to rate three vignettes. Our sample size calculation was based on data from the national GP Patient Survey, as we used the same communication questions for our respondents as are used in this national survey. Inclusion of 560 Pakistani respondents and 560 White British respondents gave over 80% power to detect a 3.1-point difference (on a 0–100 scale) seen between these two groups after controlling for age, gender, deprivation, self-rated health and practice. As ethnic disparities are largest in older ages, we aimed to recruit equal numbers above and below the age of 55 within each ethnic group.^9^

We used different recruitment strategies for the different ethnic groups. To recruit Pakistani respondents, geographically confined areas in England of ∼130 households (output areas) were selected in which at least 35% of the populations were identified as Pakistani in 2011 Census data. These were then ranked according to the proportion of the population aged over 50. Trained fieldworkers recruited participants within these areas using an in-home face-to-face approach, starting in the output areas with the highest proportion of residents over the age of 50. Available Census age categories drove our use of ‘over 50’ rather than ‘over 55’ at sampling: however, recruitment was based on an ‘over 55’ cut point. Fieldworkers were also provided with one or two neighbouring output areas to recruit from if necessary. Snowball recruitment (eg, known neighbours) and additional household interviews were allowed. To recruit White British participants, we first excluded output areas with low proportions of White British residents (<90%) and residents aged over 50. The remaining output areas were ranked by social grade (the percentage of people who were Social Grade A/B according to 2011 Census data) and geography. Output areas to approach were then selected using proportional systematic sampling.

Potential participants were screened by fieldworkers for ethnicity (using the Office for National Statistics 18-group categorisation) and English language competency (using a screening question regarding self-reported confidence in understanding short videos in English). Eligible respondents who consented then completed a computer-assisted personal interview (CAPI) using a standardised script. Each participant viewed three simulated consultation videos. Following each video, the participant was asked to rate the communication within the consultation using five items taken from the national GP–Patient Survey (table 1). Videos were assigned so each participant saw three different presenting conditions, with at least one of the videos featuring each of the two ethnic GP/patient pairings, and at least one of the videos scripted to feature each of the two levels of GP–patient communication. The selection of videos shown to each participant was such that approximately equal numbers of all possible combinations were used, given the restrictions described. Participants also completed basic sociodemographic questions (age, self-rated health, whether born in the UK, language spoken most often at home). All interview questions and ratings were completed verbally, with responses recorded by interviewers directly onto the CAPI software. An area-based measure of socioeconomic deprivation (Index of Multiple Deprivation) was calculated.

**Table 1:** GP–patient communication items

Thinking about the doctor you have just seen in the video, how good was the doctor at:											
	Very good		Good		Neither good nor poor		Poor		Very poor		Doesn't apply*
Giving enough time………………………………	□	…	□	…	□	…	□	…	□	…	□
Listening……………………………………………..	□	…	□	…	□	…	□	…	□	…	□
Explaining tests and treatments……………..	□	…	□	…	□	…	□	…	□	…	□
Involving in decisions about care ……………..	□	…	□	…	□	…	□	…	□	…	□
Treating with care and concern……………..	□	…	□	…	□	…	□	…	□	…	□

*Considered to be uninformative for the purposes of our analysis.

### Analysis

We scored each participant's rating of each consultation by linearly scaling the response options between 0 (very poor) and 100 (very good) and averaging all informative answers when at least three of the five items were completed. Linear regression was used to model the mean difference between White British and Pakistani participants' ratings of doctor–patient communication. We estimated the unadjusted difference in ratings, as well as the difference adjusting for patient age, gender, self-rated health, deprivation and a set of 15 indicator variables for the video. No analysis of interaction terms was originally planned. However, the effect size found was much larger than that anticipated, and so interactions were investigated between participant ethnicity and the following variables: (1) relating to the video: ethnicity of GP/patient and quality of GP–patient communication, and (2) relating to the participant: age, gender and deprivation. When modelling interactions, only variables for the video attributes were used (rather than using indicator variables for all videos). For interactions involving age, the oldest two age groups were combined and a continuous version of age groups was used in the interaction term only. CIs and p values were estimated using bootstrapping with 500 replications (given non-normal data), clustered by participants (with each participant supplying three communication scores). A sensitivity analysis that clustered the bootstrap resampling by output area rather than by participants (to account for multiple sampling in households and small geographic areas) made only trivial changes to SEs, so we do not report it here.

## Results

### Participants

**Figure 2:**
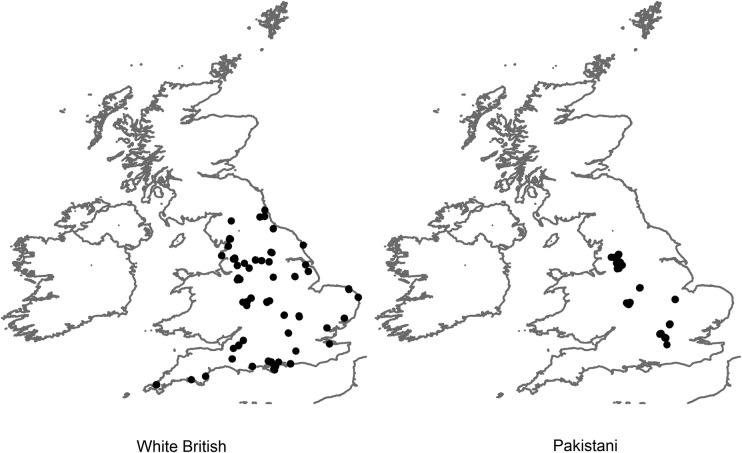
Geographic locations of the census-based output areas where White British and Pakistani participants were recruited.

A total of 1128 participants were recruited, 564 (50%) self-identified White British and 564 (50%) self-identified Pakistani participants. The sociodemographic profile of participants appears in table 2. While the sampling restriction that half of participants in each group should be aged 55 or above increased the similarity of the groups' age distribution, Pakistani participants were younger than the White British participants within each age stratum. Pakistani participants were also more likely to be man (58% vs 52%); to be in fair or poor health (38% vs 26%) and to live in the most deprived areas (82% vs 14%). The geographic locations where participants were recruited are shown in figure 2. While the White British participants were recruited from a wide range of geographic locations, the Pakistani participants were located from a small number of geographically confined locations. Between 202 and 220 participants scored each of the video vignettes for GP–patient communication (full details in online supplementary material table S1).

**Table 2:** Sociodemographic profile of study participants

	All		White British		Pakistani	
	n	Per cent	n	Per cent	n	Per cent
Age (years)						
18–24	88	7.8	40	7.1	48	8.5
25–34	154	13.7	56	9.9	98	17.4
35–44	151	13.4	70	12.4	81	14.4
45–54	175	15.5	118	20.9	57	10.1
55–64	267	23.7	94	16.7	173	30.7
65–74	179	15.9	109	19.3	70	12.4
75–84	95	8.4	63	11.2	32	5.7
85 or over	19	1.7	14	2.5	5	0.9
Gender						
Male	583	51.7	255	45.2	328	58.2
Female	545	48.3	309	54.8	236	41.8
Self-rated health						
Excellent	132	11.7	82	14.5	50	8.9
Very good	289	25.6	181	32.1	108	19.1
Good	348	30.9	157	27.8	191	33.9
Fair	207	18.4	86	15.2	121	21.5
Poor	152	13.5	58	10.3	94	16.7
Deprivation						
1—least deprived	108	9.6	100	17.7	8	1.4
2	137	12.1	137	24.3	0	0.0
3	122	10.8	111	19.7	11	2.0
4	221	19.6	138	24.5	83	14.7
5—most deprived	540	47.9	78	13.8	462	81.9

10.1136/bmjopen-2016-011256.supp1 supplementary tables

### Main results

**Figure 3:**
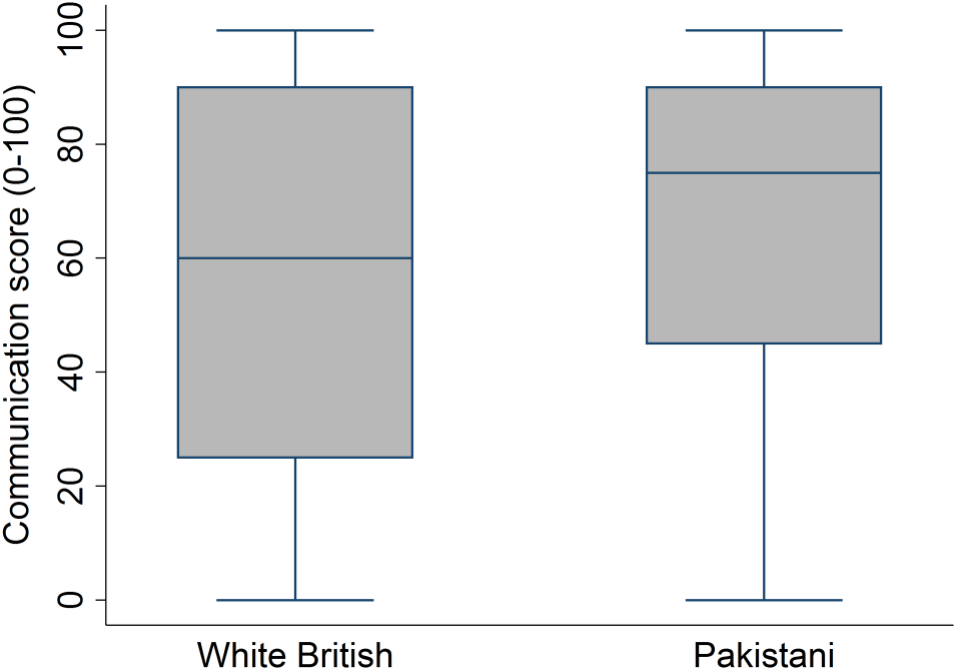
Box plots showing the distribution of GP communication scores recorded by White British and Pakistani participants.

The distribution of communication scores for White British and Pakistani participants is shown in figure 3. The data are skewed in both groups, with high communication scores given more often; however, the communication scores from Pakistani participants were typically higher than those from White British participants. The mean communication score from Pakistani participants was 67.3 out of 100, 9.9 points higher (95% CI 8.0 to 11.7, p<0.001) than the mean score from White British participants (57.4 out of 100). In a regression model (full output shown in online supplementary material table S2) adjusting for participant age, gender, self-rated health, deprivation and video, there was a slightly larger difference between the two ethnicities: 11.0 points (95% CI 8.5 to 13.5, p<0.001).

### Analysis of interactions

As the difference in scores between Pakistani and White British participants was considerably larger than that expected, we were able to explore interactions between ethnicity and other variables. There was no evidence that the difference in scores between Pakistani and White British participants varied by patient gender (p=0.92), deprivation (p=0.68) or by the ethnicity of the doctor/patient pair shown in the videos (p=0.53). There was strong evidence that the difference in scores between Pakistani and White British participants was larger for older participants (p=0.001) and consultations scripted to contain poorer doctor–patient communication (p<0.001). Table 3 shows the mean difference in age by good/poor scripted communication strata, estimated from a model containing all main effects, plus (1) ethnicity and age interactions, (2) ethnicity and good/poor communication interactions and (3) the three-way interaction between those variables (p<0.001 for three-way interaction). Interactions between presenting condition and ethnicity were omitted for clarity. The difference between scores given by younger (under 55 years) White British and Pakistani participants to consultations containing ‘good’ communication is small and not statistically significant. However, larger and statistically significant differences are seen for older patients and for consultations portraying ‘poor’ communication at all ages. In these ‘poor’ consultations, the difference in scores increases with rising age of participants. For example, ratings of consultations with poor communication are 10.29 points higher (95% CI 5.00 to 15.57) for Pakistani participants aged 18 to 24 than White British participants of the same age. This difference increases to 28.45 points (95% CI 23.11 to 33.79) for over the age of 75 years.

**Table 3:** Adjusted difference in communication scores for age group by good/poor scripted communication between White British and Pakistani participants

	Scripted communication	
Age	Good	Poor
18–24	−1.31 (−5.38, 2.76)	10.29 (5.00, 15.57)
25–34	−0.15 (−3.58, 3.27)	13.32 (9.10, 17.54)
35–44	1.01 (−1.96, 3.97)	16.34 (12.91, 19.77)
45–54	2.17 (−0.62, 4.95)	19.37 (16.24, 22.50)
55–64	3.33 (0.39, 6.27)	22.40 (18.94, 25.86)
65–74	4.49 (1.11, 7.87)	25.42 (21.16, 29.69)
75 and over	5.65 (1.64, 9.66)	28.45 (23.11, 33.79)

A positive difference implies Pakistani patients gave, on average, higher (more favourable) scores.

## Discussion

Our experimental study found that respondents from a Pakistani background rated communication in simulated GP consultations significantly more positively than their White British counterparts. These differences were largest for consultations depicting poor doctor–patient communication and for older respondents. The differences we observed were in the opposite direction to those seen repeatedly in the national GP Patient Survey, which relates to a patients' most recent consultation with a GP. In the national survey, Pakistani respondents give significantly lower scores for communication than their White British counterparts.

Our in-home face-to-face recruitment approach ensured access to a wide range of respondents, independent of the GP practice they were registered with. However, respondents who agreed to participate in this research may differ in a number of unidentified ways from the population as a whole. For example, to ensure efficient recruitment to the study, we focused our efforts on high-density Pakistani areas, which also have high levels of deprivation (the 82% of participants living in areas in the most deprived quintile compared to 51% nationally). The sampled population may, therefore, differ from the Pakistani population as a whole: for example, recent research suggests that minority ethnic populations in lower ethnic density areas may report higher satisfaction with healthcare.^18^

Our study involved face-to-face interviews in which consultations were viewed and rated: this differs from the postal mode of the national GP Patient Survey. In completing questionnaire items via an interviewer rather than independently, social desirability bias may become an issue. However, the magnitude of social desirability bias would have to be substantially different between White British and Pakistani respondents to have a large impact on our findings. Additionally, ratings of consultations by ‘analogue patients’ (members of the public asked to rate care received by a third party), such as our participants, are commonly more critical than patients commenting on their own care.^19^ In our study, across both groups, low scores were used more often than in the national GP Patient Survey: for example, only 2.6% of answers to the GP communication questions in the most recent GP Patient Survey were given as poor or very poor, compared to 26.6% of answers in this study.^20^ To enable the same vignettes to be viewed by all participants, the study was conducted in English, limiting our ability to understand evaluations by those with low English language proficiency (and who might, eg, respond to the GP Patient Survey in other languages). In the USA, ethnic minorities preferring languages other than English generally show response tendencies that are in the same direction as English-preferring members of the same ethnic minority, but to a greater extent, perhaps reflecting a continuum of acculturation.^6^ However, it was not possible to produce equivalent vignettes in other languages, and as 99.8% of respondents to the GP Patient Survey respond in English, our ability to extrapolate to the wider population remains high.

Previous examinations of inequalities in patient experience between ethnic groups have commonly relied on real-world data such as those generated through surveys, in which it is difficult to distinguish whether differences are attributable to variations in care or variations in the reporting of that care.^3–9^ Large-scale video recording of actual GP–patient consultations, an external assessment of their communication quality and the comparison of this to reported patient experiences of care would enable us to develop a more robust ‘real-world’ understanding of the drivers of variations in reported experience, but the utility of such an undertaking must be balanced against its many challenges. Our experimental design enables us to control the content of the consultations being rated by respondents in order to efficiently explore how differences in reporting may explain the disparities in minority ethnic experience in real-life surveys. We chose to focus on communication as this is a key component of quality of care, yet one where certain minority ethnic groups report consistently poor experience of their interactions with clinicians.^7–9^ The study builds on previous vignette research by using multiple video vignettes manipulating several key attributes.^12^ ^13^ Video vignettes have so far been little employed in this field, in spite of evidence of viewers perceiving them as realistic and enabling immersion in the situation at hand, although well-crafted vignettes are essential to ensure good construct validity.^14^ In the USA, Weinick *et al*, reported no evidence of differences among White, African-American and Latino evaluations of doctor–patient communication in vignettes when using an ‘Always-to-Never’ Response Scale; they concluded that variations within national surveys on such items for these groups were likely to reflect differences in real-life experiences.^13^ In our study, however, we found substantially more positive ratings by Pakistani in comparison to White British respondents.

This study was designed to explore whether people from a Pakistani background rate the communication within simulated GP consultations differently from ratings provided by White British people. Similar ratings of simulated consultations from both ethnic groups would have suggested that the low scores observed in national surveys from Pakistani respondents reflect real differences in the quality of communication experienced by these patients in comparison to White British patients. The substantially more positive ratings from Pakistani respondents that we observed in our experimental study suggest that not only are there differences in the quality of communication in real-life consultations, but also that these differences are even greater than those identified in real-life surveys, such as those we have previously reported from findings using the GP Patient Survey.^7^ ^9^ We suggest that Pakistani patients experience genuinely worse standards of communication. However, while we can be confident that differences in experience exist, it is difficult to extrapolate our vignette-derived data to estimate the magnitude of difference in real life. Poor communication for these groups may arise from system-level, provider-level and/or patient-level factors.^21^ For example, language barriers within consultations may lead to more negative experiences of care for doctors and patients.^22^ ^23^ Levels of acculturation may be linked with a patient's ability to navigate the healthcare system, with consequent impacts on patient experiences of care.^24^ Discrimination and bias are sensitive and challenging topics – whether at the level of the system or provider. However, they need to be considered as key contributors to inequalities in care.^25^

## Conclusions

Our findings add substantial weight to the likelihood that inequalities affecting South Asian people in national surveys reflect systematic variations in the quality of communication within consultations. While there is a body of research into the drivers of inequalities in care, future research needs to focus on how factors including language barriers, health literacy, provider-side discrimination and system-level failures combine to inhibit good communication within individual consultations.
