# Supplementary material for: Understanding negative feedback from South Asian patients: an experimental vignette study
Source: BMJ Open. 2016 Sep 8;6(9):e011256. doi: 10.1136/bmjopen-2016-011256 (PMC5020840; doi:10.1136/bmjopen-2016-011256)
Supplement: supplementary tables [file bmjopen-2016-011256supp_tables.pdf]

Understanding negative feedback from South Asian patients: experimental vignette study

SUPPLEMENTARY MATERIAL

Video number	Clinical scenario	Scripted communication quality	Ethnicity of Dr and patient	Number of times video scored
1	Persistent cough	Bad	White	220
2			Asian	202
3		Good	White	202
4			Asian	212
5	Perforated ear drum	Bad	White	210
6			Asian	206
7		Good	White	217
8			Asian	207
9	Painful elbow	Bad	White	206
10			Asian	210
11		Good	White	210
12			Asian	215
13	Generalised numbness	Bad	White	216
14			Asian	222
15		Good	White	212
16			Asian	214

Table 1 – Description of all video vignettes used with the number of times each video was scored

		Adjusted difference (95% CI)	P-value
Ethnicity	White British	Reference	<0.001
	Pakistani	11.01 (8.53, 13.49)	
Age (Years)	18 to 24	-5.55 (-8.94, -2.16)	<0.001
	25 to 34	-4.96 (-7.99, -1.93)	
	35 to 44	-1.67 (-4.67, 1.33)	
	45 to 54	-1.86 (-4.60, 0.87)	
	55 to 64	Reference	
	65 to 74	4.01 (1.20, 6.83)	
	75 to 84	6.70 (3.26, 10.13)	
	85 or over	3.66 (-3.66, 10.97)	
Gender	Male	Reference	0.115
	Female	1.41 (-0.34, 3.16)	
Self-rated health	Excelent	Reference	0.866
	Very good	-1.15 (-4.05, 1.74)	
	Good	-1.65 (-4.71, 1.41)	
	Fair	-1.77 (-5.12, 1.58)	
	Poor	-1.41 (-5.21, 2.38)	
Deprivation	Least deprived	Reference	0.505
	2	-0.92 (-4.10, 2.27)	
	3	1.08 (-2.26, 4.42)	
	4	-1.45 (-4.57, 1.68)	
	Most deprived	0.13 (-3.32, 3.58)	
Video number	1	Reference	<0.001
	2	-3.90 (-6.79, -1.01)	
	3	-56.51 (-60.51, -52.50)	
	4	-49.57 (-53.77, -45.37)	
	5	-4.09 (-7.06, -1.12)	
	6	-7.45 (-10.58, -4.33)	
	7	-48.08 (-51.81, -44.34)	
	8	-49.70 (-53.53, -45.87)	
	9	-3.24 (-6.33, -0.14)	
	10	-7.40 (-10.48, -4.33)	
	11	-52.19 (-56.03, -48.34)	
	12	-48.94 (-52.80, -45.08)	
	13	-9.59 (-12.89, -6.29)	
	14	-9.36 (-12.45, -6.27)	
	15	-54.23 (-58.07, -50.38)	
	16	-46.63 (-50.52, -42.73)	

Table 2 – Output from the main regression model adjusting for socio-demographic characteristics but with no interactions.
